# Supplementary material for: Polycyclic Aromatic Hydrocarbons in Indoor Dust Collected during the COVID-19 Pandemic Lockdown in Saudi Arabia: Status, Sources and Human Health Risks
Source: Int J Environ Res Public Health. 2021 Mar 8;18(5):2743. doi: 10.3390/ijerph18052743 (PMC7967472; doi:10.3390/ijerph18052743)
Supplement: Supplementary file 1 [file ijerph-18-02743-s001.pdf]

Polycyclic Aromatic Hydrocarbons in Indoor Dust Collected during COVID-19 Pandemic Lockdown in Saudi Arabia: Status, Sources and Human Health Risk

Sultan Hassan Alamri [1], Nadeem Ali [2,*], Hussain Mohammed Salem Ali Albar [3], Muhammad Imtiaz Rashid [2], Nisreen Rajeh [4], Majdy Mohammed Ali Qutub [1] and Govindan Malarvannan [5,*]

1 Department of Family Medicine, Medical College, King Abdulaziz University, Jeddah 21589, Saudi Arabia; shalamri1@kau.edu.sa (S.H.A.); mmqutub@kau.edu.sa (M.M.A.Q.)

2 Centre of Excellence in Environmental Studies, King Abdulaziz University, Jeddah 21589, Saudi Arabia; mimurad@kau.edu.sa

3 Department of Community Medicine, Medical College, King Abdulaziz University, Jeddah 21589, Saudi Arabia; hmalbar@kau.edu.sa

4 Anatomy Department, Medical College, King Abdul Aziz University, Jeddah 21589, Saudi Arabia; nrajeh@kau.edu.sa

5 Toxicological Centre, University of Antwerp, Universiteitsplein 1, Wilrijk 2610, Belgium

* Correspondence: nabahadar@kau.edu.sa (N.A.); malarvannan.govindan@uantwerpen.be (G.M.)

Table S1. Some essential characteristics of the sampled households.

1	2	3	4	5	6	7	8	9	10	11	12	13	14	15
**1**	2	no	yes	100	4	10	SC	4	yes	yes	yes (sometimes)	3	mix of all	no
**2**	3	no	yes	100	4	10	SC	4	yes	yes	no	3	mix of all	no
**3**	2	no	no	100	3	10	C	4	yes	yes	yes	3	mix of all	no
**4**	10	yes	no	120	4	20	SC	8	yes	no	no	2	F+B+S	no
**5**	3	yes	yes	120	4	30	SC	7	yes	yes	no	2	F+B+S+BK	no
**6**	7	yes	no	120	4	20	SC	7	yes	no	no	2	B+S	no
**7**	3	no	yes	100	4	10	SC	4	yes	no	no	3	mix of all	no
**8**	7	yes	yes	90	3	35	SC	6	no	yes	no	2	F+B+S+BK	no
**9**	4	no	yes	100	3	10	SC	5	yes	no	no	3	mix of all	no
**10**	1	no	yes	120	4	12	SC	4	no	yes	no	3	mix of all	no
**11**	3	yes	yes	90	3	35	SC	6	yes	yes	no	2	F+B+S+BK	no
**12**	4	yes	yes	150	5	10	NC	5	no	no	no	2	mix of all	no
**13**	10	yes	yes	140	5	2	SC	3	no	yes	sometimes	2	F+B+S+BK	no
**14**	7	yes	no	90	4	20	SC	3	no	no	no	2	F+B+S+BK	yes
**15**	1	yes	yes	90	4	10	NC	3	no	yes	no	2	F+B+S+B	yes
**16**	2	no	no	80	3	10	NC	3	yes	yes	no	3	mix of all	no
**17**	3	no	yes	120	5	10	NC	3	yes	no	no	>3	mix of all	no
**18**	1	yes	yes	80	4	4	SC	4	no	yes	no	2	mix of all	no
**19**	2	yes	yes	700	8	1	NC	5	no	yes	yes	>2	mix of all	no
**20**	5	yes	yes	80	4	10	SC	3	yes	yes	yes	2	F+B+S+BK	yes
**21**	4	yes	no	100	3	2	NC	3	yes	no	no	3	F+B+S	no
**22**	4	no	yes	80	4	10	SC	4	no	yes	no	3	mix of all	no
**23**	3	yes	no	180	5	4	NC	6	yes	yes	no	2	mix of all	no

**24**	3	yes	yes	190	5	40	NC	5	yes	no	no	3	mix of all	no
**25**	2	yes	yes	230	5	10	SC	3	no	no	no	2	mix of all	no
**26**	3	Yes	yes	120	5	15	SC	5	yes	yes	no	3	mix of all	no
**27**	2	Yes	yes	100	4	8	SC	4	yes	no	no	3	F+B+S+BK	no
**28**	5	yes	yes	140	5	10	SC	4	yes	yes	no	2	mix of all	no
**29**	4	no	no	120	5	15	SC	5	no	no	no	3	F+B+S+BK	no
**30**	3	yes	yes	100	7	12	NC	4	yes	no	no	2	F+B+S+BK	yes
**31**	5	yes	yes	300	7	7	SC	7	no	yes	no	3	F+B+S+BK	no
**32**	5	yes	yes	300	7	8	SC	7	yes	yes	no	3	mix of all	no
**33**	5	yes	yes	300	5	7	SC	7	yes	yes	yes	3	mix of all	no
**34**	4	no	yes	120	4	10	SC	4	no	no	no	2	mix of all	no
**35**	3	no	no	150	5	15	SC	4	yes	no	no	3	F+B+S+B	no
**36**	5	yes	yes	100	4	10	NC	3	no	yes	no	2	mix of all	no
**37**	7	yes	yes	120	4	15	SC	5	no	yes	no	3	mix of all	no
**38**	5	no	no	100	4	8	NC	4	no	no	no	3	mix of all	no
**39**	3	yes	yes	120	4	10	NC	4	yes	no	no	2	mix of all	no
**40**	3	yes	yes	100	3	15	SC	4	yes	yes	no	2	F+B+S+BK	no

1. Sample no.
2. Dusting /week during the lockdown
3. Indoor increase uses of incenses, e.g. Bakhoor, perfume, essential oil etc.
4. Increased use of cleaning agents (Clorox, Dettol, pesticides etc.) indoor during COVID infection
5. The total area of the house (m3)
6. Number of rooms
7. Age of the building (Years)
8. Floor-type
9. Number of people sharing the household
10. Cross ventilation with the outside through open window (yes, no)
11. Are you concerned about indoor pollution during house confinement of COVID-19?
12. Are using or taking measures to improve indoor quality, e.g. utilising an air purifier or using specific indoor plants?

13. The number of cooking per day during these days.
14. Type of cooking
15. Indoor smoking (cigarette, shisha, etc.)

*Carpeted (C)
*Semi carpeted (SC)
*Not carpeted (NC)
*Frying (F), Boiling (B), Steaming (S), Baking (BK)

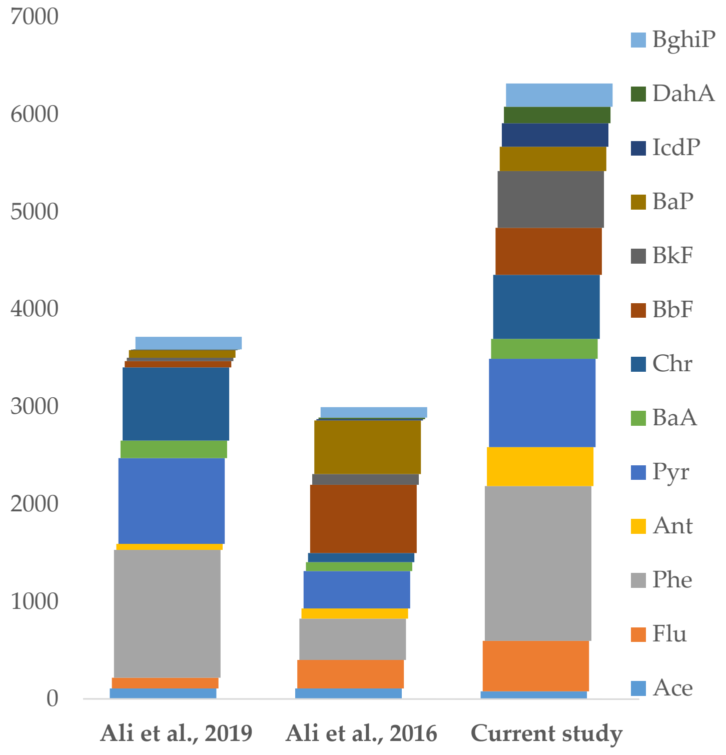

Figure S1. Profile comparison with previous studies from Jeddah, KSA
